# Fluid therapy tactics in patients with polytrauma during interhospital transportation

**DOI:** 10.1186/cc10856

**Published:** 2012-03-20

**Authors:** D Skopintsev, S Kravtsov, A Shatalin, V Agadzhanyan

**Affiliations:** 1 Federal State Budgetary Medical Prophylactic Institution, Scientific Clinical Center of Miners' Health Protection, Leninsk-Kuznetsky, Russia

## Introduction

The study's aim was to carry out a comparative evaluation of fluid therapy's influence using 130/04 hydroxyethyl (HES) starch and dextrans on hemodynamics values in patients with traumatic shock in polytrauma during interhospital transportation.

## Methods

Eighty patients with polytrauma were included in the study. Mean age was 35 ± 1 years. All patients were divided into two similar groups: experimental (EG) and control (CG). Each group was apportioned by two subgroups depending on the shock severity. Subgroup 1 consisted of patients with degree I shock, subgroup 2 comprised patients with degree II shock. The Algover-Burry index was used to evaluate the shock severity. ISS was applied to determine the injuries' severity. The injuries' severity values of the EG were the following: in subgroup 1, 25 ± 1 points; in subgroup 2, 46 ± 2 points. The values of the CG were 26 ± 1 in the first subgroup and 44 ± 2 points in the second subgroup. All patients were transported during the first 24 hours after trauma. The distance was 177 ± 9 km. The components of the fluid therapy in the CG were crystalloids and dextrans. The latter were not used in degree I shock. Crystalloid infusion was carried out on the basis of 3 ml crystalloids per 1 ml blood loss. The crystalloids and HES 130/04 starch were used in the EG. The dose of HES 130/04 starch comprised 10 to 25 ml/kg of the body mass and depended on the shock severity state. Statistical analysis was performed using Statistica 6.1. We used the Mann-Whitney criterion.

## Results

The EG patients with degree I shock had higher hemodynamics parameters (BPsys, BPdias, MAP) and less expressed tachycardia as compared to the CG patients with degree I shock (*P *< 0.05). The EG patients with degree II shock had higher hemodynamics parameters (BPsys, MAP, ESV, CI, SVR) as compared to the CG patients with degree II shock (*P *< 0.05). The change of the fluid therapy tactics in the EG resulted in the normalization of the HR, SVR and in the increase of the BPsys, MAP, ESV and CI in patients of both degrees of shock during transportation. The values of the EG were higher than in the CG during all periods of the transportation (*P *< 0.05).

## Conclusion

Inclusion of the HES 130/04 starch in the fluid therapy complex of the patients with traumatic shock in polytrauma allows one to normalize hemodynamics values at short notice and to support them adequately during all periods of transportation.

